# Spatiotemporal Gait Parameters in Fixed Versus Rotating Bearing Total Knee Arthroplasty: A Prospective 24-Month Longitudinal Study

**DOI:** 10.3390/jpm16020126

**Published:** 2026-02-21

**Authors:** Andrei Machado Viegas da Trindade, Leonardo Pinheiro Rezende, Helder Rocha da Silva Araújo, Rodolfo Borges Parreira, Claudio Santili, Claudia Santos Oliveira

**Affiliations:** 1 Department of Orthopedics Surgery, Municipal Hospital of Aparecida de Goiânia (HMAP), Aparecida de Goiânia 74936-600, Brazil; 2 Health Sciences Program, Santa Casa de São Paulo School of Medical Sciences, São Paulo 01221-020, Brazil; ortopedsantili@gmail.com (C.S.); csantos.neuro@gmail.com (C.S.O.); 3 Faculty of Medicine, University Evangelica of Goiás, Anápolis 75083-515, Brazil; leonardorezende4@gmail.com; 4 Department of Orthopaedics and Traumatology, Federal University of Goiás (UFG), Goiânia 74605-050, Brazil; drhelderrocha@hotmail.com; 5 Human Movement Analysis Laboratory, University Evangelica of Goiás, Anápolis 75083-515, Brazil; rodolfo.fcmscsp@gmail.com

**Keywords:** inertial measurement units (IMUs), gait analysis, knee, osteoarthritis, total knee arthroplasty (TKA)

## Abstract

**Background/Objectives**: The clinical superiority of rotating-bearing (RB) versus fixed-bearing (FB) total knee arthroplasty (TKA) remains controversial despite the proposed biomechanical advantages of mobile-bearing designs. Objective gait assessment with inertial measurement units (IMUs) provides a measurable view of functional recovery that may complement patient-reported outcome measures (PROMs). This study compared spatiotemporal gait parameters between FB and RB TKA over 24 months. **Methods**: This prospective longitudinal comparative study enrolled 57 patients undergoing primary unilateral TKA for end-stage knee osteoarthritis. Spatiotemporal gait parameters (gait velocity, cadence, and stance-phase duration) were measured using wireless IMUs (G-WALK system) at 6, 12, and 24 months post-surgery. WOMAC and the 10-point Geriatric Locomotive Function Scale (GLFS-10P) were assessed at 12 and 24 months. Group, time, and Group × Time effects were analyzed using linear mixed-effects models. **Results**: Both groups improved during follow-up, with performance largely plateauing between 12 and 24 months. At 24 months, there were no significant differences between groups in gait velocity (FB 1.17 vs. RB 1.16 m/s; *p* = 0.65), cadence (99.8 vs. 97.4 steps/min; *p* = 0.72), or stance-phase duration (59.3% vs. 59.0%; *p* = 0.82). Group × Time interactions were not significant across gait outcomes. WOMAC and GLFS-10P improved similarly in both groups (*p* > 0.05). Cadence was inversely correlated with the WOMAC function subscale at 24 months (rho = −0.563; *p* = 0.036). **Conclusions**: FB and RB bearing designs showed similar objective gait recovery trajectories and PROM improvements through 24 months after primary TKA, suggesting no intermediate-term functional advantage from bearing design.

## 1. Introduction

Total knee arthroplasty (TKA) is the gold standard for end-stage knee osteoarthritis, effectively restoring mobility and reducing pain [1,2]. Although 10–15-year implant survival rates exceed 90–95%, patient satisfaction and gait biomechanics often trail native knee function [1,3]. Consequently, modern research focuses on optimizing prosthetic design to minimize these kinematic and functional discrepancies.

A central debate in primary TKA concerns the choice between fixed-bearing (FB) and rotating-bearing (RB) tibial inserts [3,4]. RB designs were introduced to decouple flexion-extension from axial rotation, theoretically reducing contact stresses and facilitating physiological self-alignment [5,6]. Proponents argue that RB designs promote a more natural gait, dynamic stability, and reduced wear. Conversely, FB designs offer proven mechanical stability, reliable fixation, and a simpler surgical technique backed by decades of survivorship data [3,7].

Despite distinct theoretical approaches, clinical superiority remains debated, with inconsistent results regarding functional benefits [4]. A major methodological limitation in comparative studies is the reliance on subjective patient-reported outcome measures (PROMs) like WOMAC and the Knee Society Score (KSS) [8]. While valuable, these tools suffer from ceiling effects—particularly in successful TKA cohorts—potentially obscuring subtle kinematic differences [8]. Objective gait analysis via inertial measurement units (IMUs) offers a quantifiable, reproducible assessment of spatiotemporal parameters (e.g., velocity, cadence, symmetry) in both clinical and laboratory settings [9,10].

IMUs facilitate the detection of gait impairments that PROMs miss. However, a significant gap remains regarding prospective longitudinal studies monitoring recovery beyond 6–12 months. Extended follow-up (12–24 months) is crucial to determine if specific design features influence functional maturation during the neuromotor adaptation phase. Post-TKA gait recovery follows a biphasic pattern: rapid improvement during the first 6–12 weeks, followed by slower gains throughout the first year [11,12]. However, the long-term recovery trajectory and the specific impact of prosthesis design on this process remain underexplored.

Recent advances in wearable sensors allow for standardized gait assessment in outpatient clinics, eliminating the need for expensive motion capture labs [10,13]. Systems like G-WALK have demonstrated high validity and reliability for measuring spatiotemporal parameters across diverse patient populations [13,14]. Emerging evidence links parameters such as gait velocity and stride length to patient-perceived quality of life, underscoring their clinical relevance. Therefore, this prospective, longitudinal study aimed to compare spatiotemporal gait parameters—specifically velocity, cadence, and stance phase—between patients receiving FB and RB prostheses using validated IMU technology. In the era of personalized medicine, the goal of TKA is to match the prosthetic design to the specific phenotype and functional demands of the patient. While some argue that rotating-bearing designs offer kinematic advantages that may suit younger or more active patients, it remains unclear whether these theoretical benefits translate into measurable functional gains for the individual.

We assessed gait recovery at 6, 12, and 24 months postoperatively to determine if RB prostheses offer objective functional advantages. We hypothesized that RB designs would demonstrate superior spatiotemporal parameters and a more favorable recovery trajectory. Secondary objectives included analyzing correlations between objective metrics and PROMs (WOMAC, GLFS-10P), investigating demographic variables, and providing prospective comparative to guide prosthesis selection for primary TKA.

## 2. Methods

### 2.1. Study Design and Ethics

This prospective, longitudinal, observational study was conducted at a single tertiary referral center from 2023 to 2025. The study protocol adhered to the principles of the Declaration of Helsinki and was approved by the Institutional Review Board (IRB) of the Evangelical University of Goiás (Universidade Evangélica de Goiás—UniEVANGÉLICA).

### 2.2. Participants

Patients scheduled for primary unilateral total knee arthroplasty (TKA) due to end-stage osteoarthritis were screened for eligibility. Inclusion criteria included: (1) diagnosis of primary knee osteoarthritis; (2) indication for unilateral TKA; and (3) ability to walk independently without assistive devices before surgery. Exclusion criteria consisted of: (1) history of neurological or musculoskeletal disorders affecting gait (e.g., Parkinson’s disease, previous stroke); (2) severe osteoarthritis in the opposite knee or hip limiting mobility; (3) revision arthroplasty; and (4) inability to understand or perform the gait protocol. Patients were categorized into two groups based on the prosthetic design implanted: Fixed Bearing (FB) and Rotating Bearing (RB). The choice of implant was made by the operating surgeon based on clinical judgment and intraoperative assessment. Implant allocation was non-randomized; therefore, residual confounding and selection bias cannot be excluded. Because no randomization or matching by preoperative deformity severity was performed, between-group comparisons should be interpreted with caution, and unmeasured baseline differences may have influenced recovery trajectories.

### 2.3. Surgical Technique and Rehabilitation

All surgical procedures were performed by experienced orthopedic surgeons following a standardized protocol. A common medial parapatellar approach was used. All patients received a cemented total knee prosthesis. The surgical procedures were performed using the Modular III^®^ Fixed-Bearing (FB) system (a tibial component with a modular fixed polyethylene insert) and the Rotaflex^®^ Rotating-Bearing (RB) system (a tibial component with a mobile polyethylene insert that allows axial rotation), both manufactured by Víncula^®^ (São Paulo, Brazil). Postoperatively, all patients adhered to a standardized multimodal pain management and physical therapy rehabilitation protocol, emphasizing early mobilization, range of motion, and strengthening exercises, starting on the first postoperative day.

### 2.4. Gait Analysis Protocol

Gait assessment was conducted using a wireless inertial measurement unit (IMU) (G-WALK, BTS Bioengineering, Milan, Italy). The device, equipped with a triaxial accelerometer, gyroscope, and magnetometer, was placed at the level of the S1 vertebra using a dedicated ergonomic belt to approximate the body’s center of mass. Data collection took place at three specific postoperative time points: 6 months, 12 months, and 24 months. During the assessment, participants were instructed to perform the “Walk Test,” which involved walking along a flat, obstacle-free 10 m walkway at a self-selected, comfortable speed. To ensure data accuracy, participants completed a trial run for familiarization before the recorded trials. The raw kinematic data were processed using the proprietary software (version 1.3.0) (G-Studio, BTS Bioengineering, Italy) to determine the following spatiotemporal parameters:

- Gait Velocity (m/s): The average speed of the subject during the gait cycle.
- Cadence (steps/min): The number of steps taken each minute.
- Stance Phase (%): The percentage of the gait cycle when the foot of the operated limb touches the ground.
- Stride Length (m): The distance covered by two consecutive steps of the same limb (left and right reported separately as provided by the IMU algorithm).
- Step Length (cm): The distance between initial contacts of opposite limbs (left and right reported separately as provided by the IMU algorithm).

Prespecified outcomes: The primary outcome was gait velocity (longitudinal trajectory over 6, 12, and 24 months). Secondary gait outcomes were cadence, stance-phase duration, stride length, and step length. Exploratory outcomes included correlations between gait parameters and PROMs and subgroup analyses by age, BMI, and sex. Because implant allocation was non-randomized and surgeon-determined, between-group comparisons represent adjusted associations rather than causal effects.

Patient-reported outcome measures (PROMs) were assessed at 12 and 24 months using the Western Ontario and McMaster Universities Osteoarthritis Index (WOMAC; 24 items; total score 0–96, with lower scores indicating better outcomes; subscales: pain 0–20, stiffness 0–8, and function 0–68) and the 10-point Geriatric Locomotive Function Scale short version (GLFS-10P; 0–10, with lower scores indicating better locomotive function). PROMs were self-reported in clinic; because blinded outcome adjudication was not implemented, reporter bias cannot be excluded. Although WOMAC is widely used and facilitates comparisons with prior TKA literature, it may exhibit ceiling effects in successful TKA cohorts; future studies may consider higher-ceiling instruments (e.g., Forgotten Joint Score or High Activity Arthroplasty Score).

For clinical interpretation, WOMAC differences were contextualized using published minimal clinically important difference (MCID) values in TKA populations (typically on the order of 10–15 points, depending on the scoring scale). For GLFS-1, the scoring direction is higher = worse function; an established MCID in TKA populations is not well defined, so results are interpreted descriptively.

For the Spatiotemporal endpoints were extracted using the manufacturer’s proprietary software (G-Studio, BTS Bioengineering). To facilitate independent verification, we will make available the deidentified participant-level gait outputs used for analysis and the statistical code; the raw sensor files and software version details can be provided upon reasonable request.

### 2.5. Statistical Analysis

The primary inferential analysis used linear mixed-effects models (LMMs) to evaluate repeated measures over time. For each prespecified gait endpoint, models included fixed effects for group (FB vs. RB), time (6, 12, and 24 months; categorical), and Group × Time interaction, with participant-specific random intercepts.

- Covariate adjustment (prespecified confounders): To partially account for the non-randomized, surgeon-determined implant allocation, age, sex, BMI, and laterality were included as covariates in adjusted models.
- Estimation and reporting: We report estimated marginal means and between-group mean differences with 95% confidence intervals (CIs). The primary hypothesis test focused on the Group × Time interaction for the primary outcome (gait velocity).
- Missing data: All available observations were included via maximum likelihood estimation under a missing-at-random assumption; complete-case analyses (e.g., correlations at 24 months) were treated as exploratory and interpreted cautiously.
- Multiplicity and secondary analyses: Secondary gait endpoints and PROM endpoints were interpreted using a Benjamini–Hochberg false discovery rate (FDR) procedure (q = 0.05). Subgroup analyses by age, BMI, and sex were prespecified as exploratory (hypothesis-generating).

A two-sided alpha level of 0.05 was used. Data are presented as mean ± standard deviation (SD) for approximately normally distributed variables or median [IQR] where appropriate. CIs are provided to communicate estimation uncertainty.

## 3. Results

### 3.1. Patient Demographics

A total of 57 patients were included in the initial analysis, with 31 (54.4%) in the fixed-bearing group and 26 (45.6%) in the rotating-platform group. Baseline characteristics, including laterality distribution, are summarized in Table 1.

**Figure 1:**
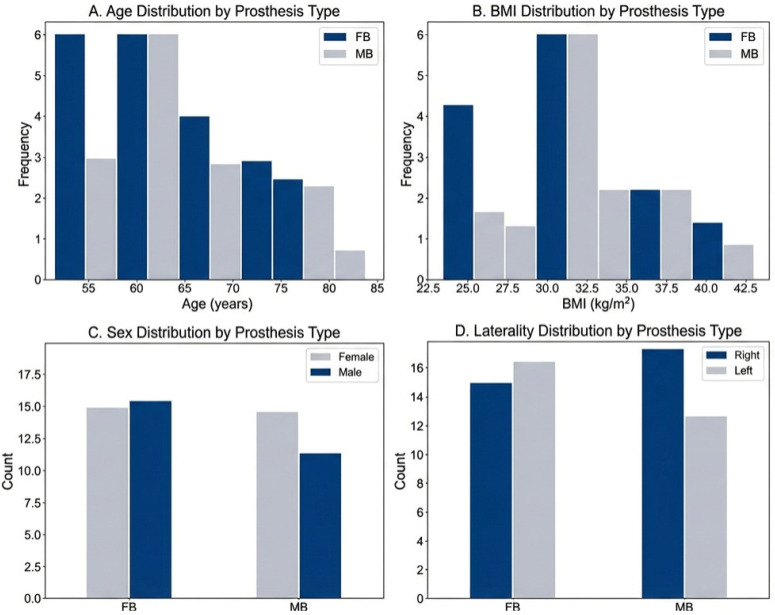
Distribution of baseline demographic characteristics across the two prosthesis groups, displaying (**A**) Age, (**B**) BMI, and (**C**) Sex., (**D**) Laterality. FB: fixed bearing; RB: rotating bearing.

Participant retention across the 6-, 12-, and 24-month assessments is summarized in the study flow diagram (Figure 1). Dropout reasons were not systematically recorded, and later follow-up estimates should therefore be interpreted with caution.

### 3.2. Between-Group Analysis of Gait Parameters (WALK Test)

**Figure 2:**
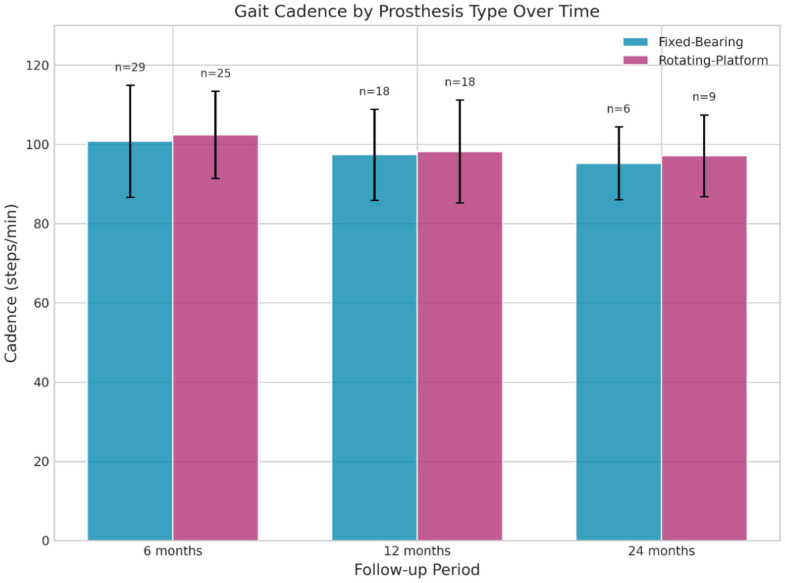
Average gait cadence for each prosthesis group at 6, 12, and 24 months postoperatively. Error bars show standard deviation. No significant differences between groups were observed. FB: fixed bearing; RB: rotating bearing.

Spatiotemporal gait endpoints were summarized at 6, 12, and 24 months. In adjusted linear mixed-effects models (group, time, and group × time; covariates age, sex, BMI, and laterality), we found no evidence of a differential recovery trajectory between FB and RB for the prespecified primary outcome (gait velocity) or secondary gait endpoints. Unadjusted per-timepoint comparisons are reported descriptively; two isolated nominal *p* < 0.05 findings were observed and should be interpreted cautiously given multiplicity and reduced complete-case sample sizes at later follow-up (Figure 2).

- At 6 months, the Fixed-Bearing group showed a slightly longer stride length on the left side compared to the Rotating-Platform group (1.17 ± 0.12 m vs. 1.10 ± 0.12 m, *p* = 0.049).
- At 24 months, the Fixed-Bearing group showed a significantly longer Step Length on the right side (52.16 ± 1.58 cm vs. 33.18 ± 24.98 cm, *p* = 0.038). The high standard deviation in the rotating-platform group at this time point indicates considerable variability. Key parameters, such as gait velocity and cadence, showed no significant differences between the groups at any follow-up time point.

### 3.3. Between-Group Analysis of PROMs (WOMAC and GLFS-10P)

**Figure 3:**
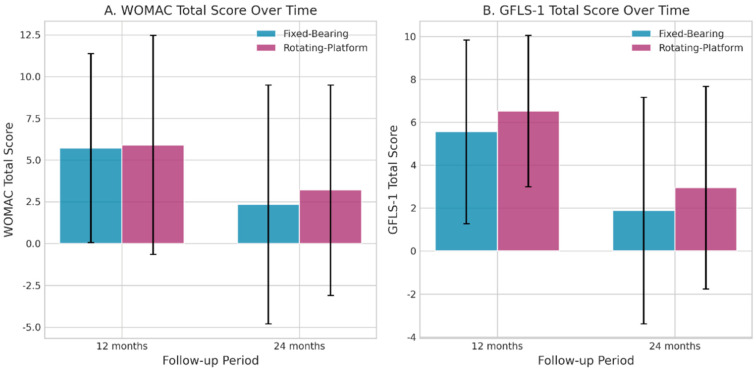
Changes in mean WOMAC Total Score and GLFS-10P Total Score from 12 to 24 months for both prosthesis groups. FB: fixed bearing; RB: rotating bearing.

Patient-reported outcomes were evaluated at 12 and 24 months (Figure 3).

- WOMAC: At 12 months, there were no significant differences in WOMAC subscales (Pain, Stiffness, Function) or total score. At 24 months, a statistically significant difference was observed in the WOMAC Total score, with the Rotating-Platform group reporting slightly better outcomes (lower scores), although the absolute difference was small (Fixed-Bearing: 2.22 ± 7.17 vs. Rotating-Platform: 2.10 ± 4.77, *p* = 0.044).
- GLFS-10P: No significant differences in the GLFS-10P total score between the two groups at 12 or 24 months.

### 3.4. Longitudinal Analysis (Within-Group Changes)

Both groups showed significant functional improvement over time.

- WALK Test: The Rotating-Platform group showed a significant decrease in cadence from 6 to 12 months (103.8 ± 12.2 vs. 98.2 ± 12.6 steps/min, *p* = 0.038). Given that cadence is influenced by self-selected speed and test conditions, this finding should be interpreted cautiously and considered descriptive. No other significant long-term changes in key gait parameters were observed.
- PROMs: Both groups exhibited significant improvements in GLFS-1 scores between 12 and 24 months (*p* = 0.030 for Fixed-Bearing, *p* = 0.026 for Rotating-Platform), reflecting enhanced locomotive function. The Fixed-Bearing group also showed a trend toward better WOMAC Total scores from 12 to 24 months (*p* = 0.053).

### 3.5. Correlation Between Gait Parameters and PROMs

**Figure 4:**
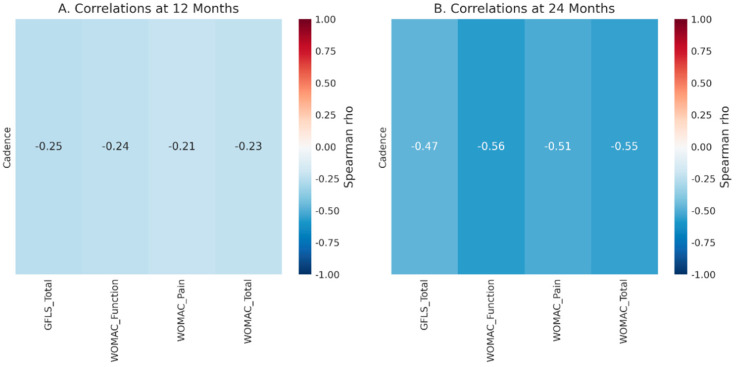
Spearman correlation heatmaps between key gait parameters and PROM scores at 12 and 24 months.

Correlation analysis was conducted to connect objective gait metrics with patients’ subjective scores (Figure 4).

- At 12 months, no significant correlations were observed between key gait parameters (Cadence, Velocity) and PROM scores (WOMAC, GLFS-1).
- At 24 months, a significant moderate negative correlation was found between cadence and WOMAC function (rho = −0.563, *p* = 0.036) and WOMAC total (rho = −0.551, *p* = 0.041). This indicates that patients with better functional scores (lower WOMAC scores) tended to have a lower cadence in this complete-case dataset.

## 4. Discussion

This prospective comparative biostatistical analysis of fixed-bearing, posterior-stabilized (PS) versus rotating-platform, ultracongruent (UC) total knee arthroplasty (TKA) showed similar functional outcomes and gait performance at 24 months. Across spatiotemporal gait metrics (WALK test), PROMs, longitudinal within-group changes, and subgroup analyses, results were consistent, indicating comparable clinical effectiveness between prosthetic designs. Most gait parameters—cadence, velocity, gait cycle duration, step length, stride length, and propulsion metrics—showed no statistically significant differences between groups at any timepoint, aligning with evidence that modern TKA designs yield convergent functional outcomes.

At 6 months, an isolated difference favored fixed-bearing for left stride length (1.17 ± 0.12 m vs. 1.10 ± 0.12 m, *p* = 0.049). However, the absolute difference (0.07 m) was small and not accompanied by differences in velocity (0.99 ± 0.14 m/s vs. 0.95 ± 0.15 m/s, *p* = 0.315) or cadence (100.79 ± 14.77 vs. 103.81 ± 12.17 steps/min, *p* = 0.409). At 24 months, fixed-bearing showed a longer right step length (52.16 ± 1.58 m vs. 33.18 ± 24.98 m, *p* = 0.038), but the large variability in the rotating-platform group (SD 24.98 m) suggests potential outliers, requiring caution.

Critically, velocity and cadence—key indicators of lower-limb function and quality of life—did not differ at any follow-up. Cadence near ~102 steps/min at 6 months and a slight decrease to ~98 at 12 months may reflect normal adaptation toward economical gait patterns [15]. Meta-analytic evidence identifies cadence, stride length, walking speed, and step length as central markers of gait recovery after TKA, with reduced double-support time indicating improved stability [16]. The neutral findings thus support the view that contemporary implants, regardless of bearing philosophy, can effectively restore walking function. PROMs improved similarly in both groups. At 12 months, WOMAC pain was comparable (1.65 ± 1.62 vs. 1.36 ± 1.76, *p* = 0.477), stiffness remained minimal, and WOMAC function did not differ (4.06 ± 4.66 vs. 5.09 ± 5.08, *p* = 0.354) [17].

At 24 months, WOMAC total differed statistically (2.22 ± 7.17 vs. 2.10 ± 4.77, *p* = 0.044), favoring rotating-platform; however, the absolute difference (0.12 points) is clinically negligible. WOMAC validity and responsiveness in TKA are well established, with typical preoperative scores ~40–60 [8,18]. Postoperative values around 2–6 in both groups imply ~90–95% improvement, independent of implant type.

GLFS-10/GLFS-based outcomes similarly showed no between-group differences at 12 months (5.56 ± 4.41 vs. 6.23 ± 3.79, *p* = 0.607) or 24 months (1.89 ± 5.43 vs. 2.30 ± 4.42, *p* = 0.514). GLFS-25 demonstrates excellent reliability (Cronbach’s alpha 0.961) and concurrent validity [19,20]. Both groups improved significantly from 12 to 24 months (fixed-bearing: *p* = 0.030; rotating-platform: *p* = 0.026), consistent with progressive neuromotor adaptation and reduced joint awareness over time [16,21,22]. The agreement between objective gait and subjective outcomes reinforces that prosthetic design is not a primary driver of function in primary TKA when implantation and balancing are adequate.

Within-group analysis showed meaningful recovery trends. Rotating-platform cadence decreased significantly from 6 to 12 months (103.81 ± 12.17 to 98.19 ± 12.62, *p* = 0.038), with a similar non-significant trend in fixed-bearing (100.79 ± 14.77 to 97.36 ± 11.13, *p* = 0.210). This likely reflects adoption of more stable, economical gait strategies rather than decline [12,16]. PROMs also improved from 12 to 24 months, with significant GLFS-10p reductions (fixed-bearing: 5.56 ± 4.28 to 1.89 ± 5.28, *p* = 0.030; rotating-platform: 6.52 ± 3.53 to 2.95 ± 4.72, *p* = 0.026), matching the biphasic recovery model after TKA (early rapid gains, then slower continued improvements through 12–24 months) [7,11,12].

At 24 months, cadence correlated moderately and negatively with WOMAC function (rho = −0.563, *p* = 0.036) and WOMAC total (rho = −0.551, *p* = 0.041), while no significant correlations were present at 12 months. This suggests that lower cadence may reflect more controlled, efficient gait associated with better perceived function, highlighting that gait quality may matter more than stepping frequency alone [16,21]. This supports multimodal outcome assessment rather than relying on isolated spatiotemporal variables [16].

Demographic stratification (age, BMI, sex) showed no significant interactions, indicating no consistent design advantage in specific cohorts [4,23,24]. Prosthesis selection should therefore prioritize surgeon expertise and patient-specific anatomy rather than demographic optimization. Our findings have significant implications for personalized medicine in orthopedics. The absence of superior gait parameters in the RB group suggests that functional recovery is less dependent on the bearing mobility mechanism and more reliant on patient-specific factors and rehabilitation. In a personalized care model, this allows the surgeon to select the implant based on individual anatomical needs—such as using a Fixed-Bearing design for a patient requiring greater stability due to ligament laxity, or a Rotating-Bearing design to potentially reduce polyethylene wear in a younger patient—without the concern of compromising spatiotemporal gait recovery.

These results align with broader evidence. A systematic review/meta-analysis comparing mobile- versus fixed-bearing TKA found no significant differences in revision, aseptic loosening, functional scores, flexion, or radiographic outcomes across short-, mid-, and long-term follow-up [4]. A 12-year prospective randomized trial similarly found no differences in Knee Society Scores, WOMAC, SF-36, satisfaction, or survival, with high long-term success probabilities for both implants [3]. Evidence comparing PS versus cruciate-retaining designs in genu recurvatum suggests PS may improve flexion and reduce hyperextension recurrence without differences in overall function, satisfaction, or revision, underscoring individualized selection based on intraoperative findings [23].

Rehabilitation appears more influential than implant philosophy. High-intensity and early mobilization approaches improve strength and function without compromising pain or ROM [7,11,12]. APTA clinical practice guidelines recommend early mobility planning and motor function training (balance, walking, symmetry) with strong evidence [25], and early PT initiation (within 24 h, including ultra-early within 12 h) may reduce length of stay without harming outcomes [12]. Clinically, the absence of meaningful differences supports selecting implants based on anatomy, deformity, ligament competence, and surgeon familiarity. PS designs may be preferable when PCL deficiency, flexion contracture, or severe deformity requires predictable stability [6,7,23], whereas rotating-platform designs may suit deep-flexion demands or durability concerns in younger/high-activity patients [3,4].

Strengths include a prospective comparative design, 24-month follow-up capturing functional stabilization, objective gait assessment combined with validated PROMs, and standardized instruments with established psychometrics [8,17,18,19,20]. Subgroup analyses across age, BMI, and sex supported generalizability. The use of non-parametric methods, Wilcoxon paired testing, and Spearman correlations reflects appropriate biostatistical practice.

Key limitations include limited power (57 patients), absence of long-term durability endpoints (10–15 years), and insufficient detail on baseline deformity, ROM, comorbidities, and surgical technique. The lack of radiographic alignment, component positioning, and wear markers limits mechanistic interpretation. Rehabilitation protocol detail and adherence data were not reported, and satisfaction/complications were not fully addressed. Correlation analyses relied on complete-case subsets (n = 14 at 24 months), raising risks of low power, multiple comparisons, and potential type I error without correction. Attrition across follow-up also suggests possible attrition bias.

Future work should include long-term follow-up (≥10 years) with radiographic, wear, and revision endpoints. Randomized trials with advanced biomechanics (3D gait analysis, kinematic fluoroscopy) and neurophysiologic measures could clarify mechanisms. Studies should directly test rehabilitation intensity and neuromuscular training as primary drivers, using implant type as a stratifier. Research on expectations, shared decision-making, and satisfaction trajectories is needed, as are health economic analyses incorporating implant costs, revision risk, and quality-adjusted life years.

## 5. Conclusions

In conclusion, this prospective comparative study found similar spatiotemporal gait recovery trajectories and PROMs between fixed-bearing and rotating-bearing posterior-stabilized TKA through 24 months. Any between-group differences were small and inconsistent and did not suggest clinically meaningful superiority of either bearing design. Given the non-randomized allocation and declining follow-up completeness, these findings should be interpreted cautiously and validated in larger, adequately powered studies incorporating preoperative baseline gait assessment and steady-state walking protocols.

## Figures and Tables

**Table 1:** Demographic and Baseline Characteristics of Study Participants.

Variable	Fixed-Bearing (*n* = 31)	Rotating-Platform (*n* = 26)	*p*-Value	Test
Age (years)	68.4 ± 8.2	68.0 ± 7.4	0.841	Student’s t-test
BMI (kg/m^2^)	30.3 ± 5.1	31.2 ± 4.8	0.498	Student’s t-test
Weight (kg)	78.9 ± 13.6	78.4 ± 13.8	0.754	Mann–Whitney U
Height (m)	1.61 ± 0.10	1.59 ± 0.09	0.286	Mann–Whitney U
Sex (Female/Male)	19/12 (61.3%/38.7%)	16/10 (61.5%/38.5%)	1.000	Chi-square
Laterality (Right/Left)	16/15 (51.6%/48.4%)	17/9 (65.4%/34.6%)	0.436	Chi-square
Age group (<65/≥65 years)	10/21 (32.3%/67.7%)	9/17 (34.6%/65.4%)	1.000	Chi-square

## Data Availability

The original contributions presented in this study are included in the article. Further inquiries can be directed to the corresponding author.
